# Co-creation of a novel approach for improving supply chain management for SARS-CoV-2 point of care diagnostic services in Mopani District, Limpopo Province: nominal group technique

**DOI:** 10.3389/fpubh.2024.1378508

**Published:** 2024-05-09

**Authors:** Kuhlula Maluleke, Alfred Musekiwa, Siphesihle Nxele, Boitumelo Moetlhoa, Langa Makena, Nkosingiphile Nzuza, Alarice Lenders, Ncomeka Manentsa, Tiyiselani Maswanganyi, Thobeka Dlangalala, Tivani Mashamba-Thompson

**Affiliations:** ^1^School of Health Systems and Public Health, Faculty of Health Sciences, University of Pretoria, Pretoria, South Africa; ^2^Limpopo Department of Health, Pharmaceutical Directorate, Polokwane, South Africa; ^3^Limpopo Department of Health, Laboratory and Blood Services, Polokwane, South Africa; ^4^National Department of Health, HIV Prevention Directorate, Pretoria, South Africa; ^5^Ezintsha, Faculty of Health Sciences, University of the Witwatersrand, Johannesburg, South Africa; ^6^North-West Department of Health, Pharmaceutical Services, Mmabatho, South Africa; ^7^Faculty of Health Sciences, University of Pretoria, Pretoria, South Africa

**Keywords:** nominal group technique, co-creation, supply chain management, point-of-care diagnostic (POC), COVID-19

## Abstract

**Introduction:**

Effective supply chain management (SCM) of point-of-care (POC) tests for diseases like severe acute respiratory syndrome coronavirus type 2 (SARS-CoV-2) requires active participation from diverse stakeholders, government entities, and regulatory bodies. The responsibility for overseeing various aspects of POC tests, including procurement, quality assurance, storage, inventory management, distribution, and human resource capacity, lies with national, provincial, and local levels of government. This study aimed to collaboratively develop an innovative approach to enhance SCM for SARS-CoV-2 POC diagnostic services in resource-limited settings, using the Mopani District in Limpopo province, South Africa, as a case study.

**Methods:**

Key stakeholders were invited to participate in an online workshop using purposive sampling. The study employed the nominal group technique (NGT) for data collection, which consisted of two phases. Phase 1 focused on identifying barriers in the supply chain of COVID-19 rapid tests, while phase 2 aimed to devise strategies to overcome the priority barriers identified in phase 1. Participants used a Likert scale of 1–5 to rank barriers and strategies, and an overall ranking score was calculated for each. The participants were provided with the results of the ranking exercise for their feedback.

**Results:**

Eleven key stakeholders from national (*n* = 1), provincial (*n* = 4), and local government (*n* = 2) levels, research entities (*n* = 3), and non-governmental organizations (*n* = 1) took part in the study. Participants identified significant barriers in the supply chain, such as the availability of testing kits, unknown demand, information on SCM during a pandemic, methods of controlling stock, and procurement processes. Strategies suggested by key stakeholders included monitoring stock levels and optimizing stock visibility systems to improve test availability, enhancing information visibility and consistent data updates to address unknown demand and improve SCM during a pandemic, employing data capturing and digitization for effective stock control, and implementing demand planning and standardized procurement processes at the national level to enhance stock procurement.

**Discussion:**

The successful collaboration with key stakeholders, facilitated by the NGT, resulted in the co-creation of a novel approach to enhance SCM for COVID-19 diagnostic services in resource-limited settings. This study holds the potential to support the provision of COVID-19 diagnostic services in such settings. A recommended follow-up study would assess the feasibility of implementing this approach.

## 1 Introduction

The healthcare sector’s supply chain management (SCM) systems face vulnerabilities due to the intricate nature of the coronavirus disease 2019 (COVID-19), fluctuations in demand for specific commodities, and the criticality of dealing with human lives (1). SCM entails regulating the flow of medical and essential goods and services from manufacturers to patients (1, 2), and it remains a complex and fragmented process, irrespective of the outbreak’s severity (3). Unprecedented measures were necessitated by the government to prevent or mitigate risks associated with the COVID-19 outbreak, particularly in the supply and distribution of point-of-care (POC) tests crucial for controlling and managing COVID-19 (1, 4, 5).

The involvement of various stakeholders, including government institutions and regulatory agencies, is imperative in SCM for POC tests (6, 7). The government bears the responsibility for administering SCM of POC tests to primary healthcare (PHC) clinics, encompassing procurement, quality assurance, storage, distribution, and human resource capacity (8, 9). The provincial government, funded by the national government, must adhere to a comprehensive procurement process, ensuring value for money without compromising on the quality of procured POC tests (10).

The SCM of COVID-19 rapid tests in Mopani District underwent two phases. The first phase witnessed emergency procurement processes, a lack of standardization, and deficient stock management (11, 12). Emergency procurement was necessitated due to a lack of prior knowledge about the disease (13), and an international organization donated stock, leading to inconsistent stock management and manual control at Van Velden hospital, the distribution centre. Reporting was suboptimal, with only 57.6% of facilities utilizing the CSA reporting application (14). In the second phase, starting in 2021, the pharmaceutical depot assumed SCM control. However, the absence of historical data on consumption from phase 1 hindered the determination of appropriate POC test quantities for each facility (15). Adopting a push allocation model resulted in overstocking and equal distribution to all PHC clinics, regardless of usage (16). District hospitals implemented disparate approaches to stock delivery to PHC clinics based on their resources, leading to unequal distribution and depriving some PHC clinics with higher usage.

This study’s primary objective was to collaboratively develop an acceptable SCM approach for enhancing SARS-CoV-2 POC diagnostic services in resource-limited settings, utilizing Mopani District in Limpopo province, South Africa, as a study setting. The anticipated outcome is that the study results will contribute to establishing a sustainable SCM approach for improving SARS-CoV-2 diagnostic services in resource-limited settings.

## 2 Materials and methods

### 2.1 Study setting

Mopani District municipality, situated in Limpopo province, South Africa, comprises 16 urban areas and 354 villages, with a population of 1,202,916 (17). The district is divided into five subdistricts, namely Greater Giyani, Ba-Phalaborwa, Greater Letaba, Greater Tzaneen, and Maruleng. Despite its size, the district faces service delivery challenges, reporting lower averages than national averages in providing basic health care services (18).

### 2.2 Study population

Key stakeholders for this study are defined as individuals, groups, or organizations who have a direct interest, expertise, or involvement in the SCM of POC diagnostic services. These stakeholders include: healthcare providers (medical professionals and laboratory technicians) involved in the delivery of COVID-19 healthcare services; government employees (officials and policymakers from local, provincial, and national government departments) responsible for healthcare, public health, and procurement; non-governmental organizations (NGOs) (humanitarian agencies, advocacy groups, and health-focused NGOs) which provide support, resources, or advocacy for improving SCM of diagnostic services; and research entities (academic institutions, research organizations, or research-focused departments within government agencies) engaged in studying healthcare systems, epidemiology, or public health interventions, and contributing to the development of evidence-based strategies for SCM improvement.

### 2.3 Study design

This study is part of a multi-phase investigation focused on developing a novel approach to improve SCM for SARS-CoV-2 POC diagnostic services in resource-limited settings, specifically in Mopani District Municipality, Limpopo, South Africa (19). The study was guided by the lean and agile SCM conceptual framework, further enhanced by guidance from the World Health Organization (WHO) manual for procurement of diagnostics and associated laboratory items and equipment (9, 20, 21). The study utilized a mixed methods approach across four phases. Phase 1 entailed a scoping review, which underscored the criticality of equitable access to diagnostic tests at POC through well-coordinated SCM systems, particularly in settings with constrained access to diagnostic laboratory services (22). Phase 2, comprising geospatial analysis, revealed that a substantial proportion (78.2%) of the population had sufficient accessibility to COVID-19 diagnostic services, presuming utilization of the nearest healthcare facility; however, it identified an uneven distribution of diagnostic services within the region. Phase 3 involved an audit of POC diagnostic services, exposing non-compliance with SCM practices in PHC clinics in the Mopani District, notably pertaining to procurement challenges, inadequate inventory management, substandard storage facilities, disjointed distribution, deficient personnel training, and poor adherence to quality assurance standards (23). These findings prompted Phase 4, the nominal group technique (NGT) investigation, involving a collaborative process with key stakeholders to devise an innovative approach addressing the identified SCM challenges. NGT, a qualitative study modality characterized by structured group interaction among participants, facilitates the collective sharing of perspectives to enhance policy and other public health-related issues (24, 25). It ensures equitable contribution from all participants, thereby preventing the dominance of ideas by any single individual (26).

### 2.4 Sampling

Purposive sampling was employed to ensure representation from national, provincial, and local levels of government, research entities, and non-governmental organizations. An online nominal group discussion using the Zoom platform included at least one participant from each identified stakeholder category. A sample size of eleven participants was determined through careful consideration of the team’s expertise and representation of diverse perspectives pertinent to the research question. In establishing the number of participants for the nominal group workshop, we drew upon recommendations from existing literature. Previous research suggests that the range of participants for NGT workshops varies from 5 to 16 individuals, with an average group size typically consisting of 7–10 experts (27). The preferred group size falls within the range of 6–12 participants, as advocated by scholarly sources (28).

#### 2.4.1 Inclusion criteria

The nominal group technique included participants who met the following criteria:

- Key stakeholders involved in the SCM of COVID-19 POC diagnostic services.
- Individuals with expert knowledge in SCM of POC diagnostic tests.
- Individuals with an interest in implementing efficient SCM processes for POC diagnostic tests.
- Representation from healthcare providers, government employees, non-governmental organizations, and research entities.

#### 2.4.2 Exclusion criteria

The nominal group technique excluded participants based on the following criteria:

- Individuals lacking expertise or experience in SCM of POC diagnostic tests.
- Individuals not involved or interested in implementing efficient SCM processes for POC diagnostic tests.
- Participants outside the specified stakeholder categories.

### 2.5 Recruitment strategy

Invitations were sent via email to the identified participants, outlining the purpose of the stakeholder engagement and the platform created for discussing innovative approaches to improving SCM of COVID-19 POC diagnostic services.

### 2.6 Nominal group technique process

The convened stakeholders participated in an online workshop on March 1, 2023, utilizing the NGT for data collection. The workshop consisted of two phases, employing a structured group discussion format to establish consensus on priorities in response to the research questions.

#### 2.6.1 Phase 1: identification of barriers

Participants, communicating in English, were prompted to answer the following question: “What are the barriers/challenges faced in the supply chain of COVID-19 rapid tests in Mopani District?” The following steps were undertaken:

Silent Brainstorming: Participants were given 10 min for silent brainstorming, documenting responses without discussion. Clarification requests were indicated by raising hands for facilitator attention.
Round Robin Session: A 20 min allocation allowed each participant to present ideas in a round-robin manner. Ideas were categorized into themes for subsequent presentation and discussion.
Discussion and Clarification: The facilitator, assisted by an assistant, collated and highlighted similar themes. Participants engaged in discussion, seeking clarifications and probing presenters. Collated results were presented to the group for ranking during the subsequent session.
Ranking of Ideas: A ranking process involved assigning values to ideas based on priority (29). Participants had a break, during which a Google Form was created for the ranking stage of NGT. Using a Likert scale (1–5), participants independently ranked barriers’ priority. Results were collated and analysed using an Excel spreadsheet.

#### 2.6.2 Phase 2: proposed Strategies

The facilitator posed the question: “What are the strategies to overcome the top barriers or challenges?” This phase mirrored Phase 1 steps, with discussions and clarifications leading to the final stage of ranking priority strategies. The same Likert scale (1–5) was utilized, and results were collected and analysed following a similar approach.

### 2.7 Data collection

The NGT was conducted in two sequential phases. Phase 1 involved the identification of barriers, while Phase 2 was dedicated to proposing strategies to address the identified barriers. Each phase consisted of the following sub-phases:

- Silent generation of ideas: Participants independently generated ideas in response to a posed question, documenting them on paper.
- Round-robin sharing: Participants took turns sharing their responses without engaging in discussion or critique. These responses were systematically listed on the chat box. This process continued until all participants had shared their responses.
- Discussion phase: Group members participated in structured discussions, posing questions to clarify list items and elaborate on their responses. Similar meanings were amalgamated, and duplicate items were subject to removal during this phase.
- Voting phase: Each participant assigned ranks to prioritize the listed items. The ranking results were then compiled to form a consolidated list of priorities for the broader group using a Likert scale of 1–5, with 1 representing low priority and 5 representing high priority.

### 2.8 Data management

The data collection encompassed two distinct types: quantitative and qualitative. To ensure methodological rigor and facilitate comprehensive analysis, we adopted specific tools for each data type, subsequently integrating outcomes to address our research inquiries.

#### 2.8.1 Quantitative data

For the quantitative aspect, we employed Microsoft Excel spreadsheets to systematically organize and manage the numerical data derived from the nominal group discussions. This methodical approach allowed us to conduct statistical analyses, identify patterns, and discern trends in stakeholder priorities (30). The quantitative data primarily comprised the rankings assigned by participants to various barriers and proposed strategies.

#### 2.8.2 Qualitative data

During the discussions, qualitative data were diligently recorded on the Zoom platform and in a dedicated notebook to preserve the richness and depth of stakeholder insights. This qualitative dataset served as a valuable resource for in-depth analysis, interpretation, and thematic identification. The recorded information encompassed participants’ ideas, comments, and discussions related to barriers and proposed strategies. Subsequent to the nominal group discussions, key stakeholders further enriched our dataset by providing additional qualitative data. These valuable comments were received in response to the workshop report disseminated promptly after the event. The incorporation of these insights served to enhance the depth of our analysis, capturing perspectives beyond the confines of the workshop setting.

### 2.9 Data analysis

Participants assigned numerical scores to each barrier, reflecting their perceived level of importance. These individual scores were then aggregated to calculate a comprehensive total importance score for every identified barrier. The scoring scale ranged from 1 to 5, with 1 denoting low priority and 5 indicating high priority. Following the identification of the top 5 barriers in Phase 1, the subsequent phase focused on proposing strategies to overcome these challenges. Similar to the first phase, a quantitative approach was adopted to evaluate and rank the effectiveness of these strategies. Each strategy received a total importance score based on its perceived effectiveness in addressing the identified barriers. Participants provided scores within the same 1 to 5 scale, where 1 indicated low priority and 5 signified high priority. The total importance scores assigned by individual participants were aggregated to determine the overall effectiveness ranking of the proposed strategies.

### 2.10 Ethical considerations

Ethical approval to conduct this study was granted by the University of Pretoria Faculty of Health Sciences Research Ethics Committee (Reference No: 655/2021, Dated: 24 November 2021) and the Limpopo Department of Health ethics committee (Reference No: LP_2021-12-007, Dated: 27 February 2022). Additional written permission was obtained from the Mopani District Department of Health. Prior to their involvement, all participants received comprehensive information about the study’s background, objectives, and methodology. The researcher addressed any queries or concerns raised by participants regarding the study. Moreover, participants provided informed consent by signing consent forms, indicating their voluntary participation in the workshop. To maintain confidentiality, the identities and personal information of all participants were kept secure, and all information shared during the discussions was anonymized. Furthermore, in consideration of the recorded sessions, a password protection and encryption were implemented to ensure the security and confidentiality of the recordings.

## 3 Results

### 3.1 Characteristics of participants

Eleven key stakeholders actively engaged in the supply chain management (SCM) of COVID-19 rapid tests in Mopani District participated in the workshop, with ages ranging from 26 to 55. A notable gender distribution was observed, with 10 participants (90.9%) being female. The majority of participants (72.7%) were employed, and among them, two (18.2%) served as post-doctoral research fellows, while one (9.1%) held the position of a full-time PhD candidate. Additional and detailed characteristics, along with the specific roles of the participants in SCM, are presented in Table 1.

**Table 1:** Characteristics of participants.

Gender	Age (years)	Occupation	Stakeholder group	Role in SCM
Female	26–35	Pharmacist	Provincial government	Managing dispensing of COVID-19 rapid tests
Female	36–45	Program Lead	National government	Supporting the acceleration of POC rapid testing
Male	46–55	Pharmaceutical depot manager	Provincial government	Overseeing the distribution of COVID-19 rapid tests to all districts in the province
Female	36–45	Pharmacist & Warehouse manager	Provincial government	Coordinating the receiving and distribution of COVID-19 rapid tests and maintain accurate inventory records
Female	36–45	Laboratory & Blood Service Deputy Director	Provincial government	Coordinating laboratory and blood services
Female	26–35	Medical Doctor & Research Clinician	NGO	End-user (COVID-19 testing) and generation of new knowledge by conducting clinical trials
Female	36–45	Post-doctoral research fellow	Research entity	Conducting research of optimal implementation of POC diagnostics services
Female	26–35	Post-doctoral research fellow	Research entity	Conducting research of optimal implementation of POC diagnostics services
Female	26–35	PhD candidate	Research entity	Conducting research of optimal implementation of POC diagnostics services
Female	46–55	Professional Nurse & Mopani District Public Health	Local government	Coordinating disease control unit & facilitate distribution of COVID-19 rapid tests
Female	46–55	Professional Nurse	Local government	End-user (COVID-19 testing)

### 3.2 Quantitative findings

#### 3.2.1 Phase 1: stakeholders’ perspective on the barriers faced in the supply chain of COVID-19 diagnostic services in Mopani District

**Figure 1:**
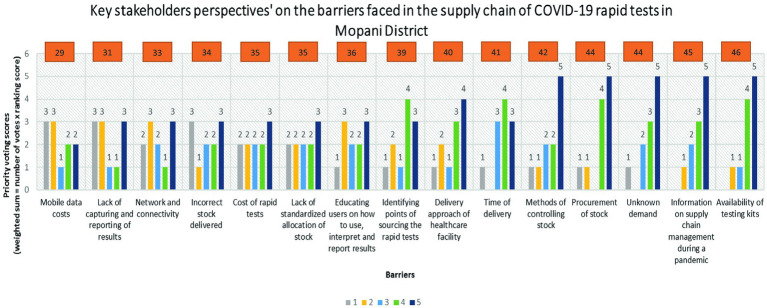
Key stakeholders’ voting scores on the barriers faced in the supply chain of COVID-19 rapid tests in Mopani District.

The stakeholders identified 15 distinct barriers within the supply chain of COVID-19 rapid tests in the Mopani District. Figure 1 presents the prioritization of these barriers, depicted in ascending order based on their respective priority scores. According to the voting results, the highest priority was assigned to the availability of testing kits, receiving a score of 46. Subsequently, information on supply chain management during a pandemic followed closely with a score of 45, along with unknown demand and procurement of stock receiving scores of 44. Other prioritized barriers included methods of controlling stock (score of 42), time of delivery (score of 41), and the delivery approach of healthcare facilities (score of 40). Conversely, mobile data costs emerged as the least severe barrier, garnering a score of 29. This was succeeded by challenges such as lack of capturing and reporting of results (score of 31), network and connectivity issues (score of 33), incorrect stock delivered (score of 34), cost of rapid tests (score of 35), educating users on result-related procedures (score of 36), and identifying points of sourcing rapid tests (score of 39).

#### 3.2.2 Phase 2: proposed potential strategies to overcome the barriers to SCM for COVID-19 rapid tests

Table 2 presents the 28 proposed potential strategies suggested by participants to overcome the five highly ranked barriers in the supply chain of COVID-19 rapid tests in Mopani District, which are availability of testing kits, information on SCM during a pandemic, unknown demand, procurement of stock, and methods of controlling stock. The participants ranked these strategies based on their effectiveness to overcome the identified barriers.

**Table 2:** Proposed strategies to overcome the top five barriers to SCM of COVID-19 rapid tests.

Proposed strategies to improve the top 5 barriers to SCM of COVID-19 rapid tests	Summing by votes 1 = less effective 5 = highly effective					Total number of voting scores (weighted sum = number of votes × ranking score) 55
	1	2	3	4	5	
Availability of testing kits						
Policies to be put in place on a national level	1	2	2	1	5	40
Buffer stock to be provided		1	5	2	3	40
Understanding needs of community healthcare facilities	1		3	3	4	42
Managing processes from procurement to end user			2	5	4	46
**Monitoring of stock levels**				**2**	**9**	**53**
**Optimization of stock visibility system (SVS)**				**1**	**10**	**54**
Unknown demand						
Forecasting demand review to draw up historical data for demand planning			2	6	3	45
Feedback from healthcare facilities			1	4	6	49
**Visibility and information**			**1**	**3**	**7**	**50**
**Consistent update of data capturing systems**				**4**	**7**	**51**
Information on SCM during a pandemic						
National Treasury to provide instruction notes	1	1	3	3	3	39
Strategies to be provided to communities at large from NDoH		1	2	6	2	41
Standard guidelines to be provided	1		2	4	4	43
**Policies to be put in place on a national level**		**1**	**2**		**8**	**48**
**Reporting what facilities have received and consumed**			**1**	**3**	**7**	**50**
Methods of controlling stock						
Apps should be free		1	1	2	7	48
Educating users on using and interpreting data			2	3	6	48
Availability of stock to be reported				6	5	49
Weekly counts to check stock				6	5	49
**Capturing data**			**1**	**3**	**7**	**50**
**Digitization**			**1**	**3**	**7**	**50**
Procurement of stock						
Provinces should have personalized procedures specific to each province	1		3	4	3	41
Understand needs of community healthcare facilities		1	1	3	6	47
Policies to be put in place on a national level		1		4	6	48
What is to be procured must be clear			1	5	5	48
**Demand planning to be in place**			**2**	**2**	**7**	**49**
**Standardized procurement procedures**		**1**	**1**	**1**	**8**	**49**

The key stakeholders ranked monitoring of stock levels (96.4%) and optimization of stock visibility systems (SVS) (98.2%) as the most effective strategy to improve the availability of COVID-19 rapid tests. To improve the unknown demand, visibility of information (90.9%) and consistent updating of data capturing systems (92.7%) were ranked as the most effective strategies. For improving information on SCM during a pandemic, putting policies in place at the national level (87.3%) and reporting on facilities that have received and consumed supplies (90.9%) were the most effective strategies. Capturing of data (90.9%) and digitization (90.9%) were ranked as the most effective methods of controlling stock. To improve the procurement of stock, participants suggested that demand planning should be in place (89.9%) and standardized procurement procedures (89.9%).

### 3.3 Qualitative findings

Given that Supply Chain Management (SCM) functions as an interconnected system wherein the efficacy of the entire system hinges on the optimal performance of each constituent, we meticulously examined the interplay between the prominently identified barriers and the suggested strategies by the stakeholders. Table 3 serves as an illustrative framework aligning SCM barriers with proposed strategies, elucidating three overarching themes: digitization, visibility of information, and procurement policies. Six out of the ten proposed strategies focalize on digitizing SCM systems, with a specific emphasis on enhancing the Stock Visibility System (SVS). These strategies encompass activities such as monitoring stock levels, consistent updates of data capturing systems, data capture, reporting of received and consumed facilities, demand planning, and optimizing the SVS. The digitization initiatives are poised to establish a foundation for real-time data acquisition, enabling vigilant stock monitoring and data-driven demand planning. Meanwhile, two of the ten proposed strategies are intricately tied to procurement policies, specifically advocating for the establishment of national-level policies and the standardization of procurement processes. Soliciting input from all 11 key stakeholders, we sought comments and additional suggestions on implementing the proposed potential strategies for enhancing the SCM of SARS-CoV-2 diagnostic services. The subsequent sections delineate these themes, offering insights into the participants’ qualitative comments and suggestions.

**Table 3:** Matching the SCM barriers with the proposed strategies for improving SCM of COVID-19 rapid tests.

Barriers	Proposed strategies	Theme
Availability of testing kits	Monitoring of stock levels	Digitization
	Optimization of SVS	Digitization
Unknown demand	Visibility of information	Visibility of information
	Consistent update of data capturing systems	Digitization
Information on SCM during a pandemic	Policies to be put in place at national level	Procurement policies
	Reporting what facilities have received and consumed	Digitization
Methods of controlling stock	Capturing data	Digitization
	Digitization	Digitization
Procurement of stock	Demand planning to be put in place	Digitization
	Standardize procurement processes	Procurement policies

### 3.4 Digitization

The incorporation of digitization as a central theme is underscored by key qualitative insights, emphasizing its role in augmenting stock level monitoring, specifically through optimizing the Stock Visibility System (SVS). One participant emphasized the seamless integration of rapid tests into the SVS’s listed items, offering comprehensive and immediate insights into test quantities received, utilized, and currently available at each facility.

“Currently, Stock Visibility System is the only means of monitoring stock that is available in our country. We need to get the rapid test to be part of the Stock Visibility System listed items so that at a click of a button, all stakeholders will know the number of tests the facility received, the number of tests the facility used and the number of tests they have at hand.”

Another strategic proposition highlighted the integration of SVS with complementary systems, such as electronic health records, providing a comprehensive view of inventory levels and patient needs.

“The government must integrate SVS with other systems like electronic health records to provide a more holistic view of inventory levels and the patient needs.”

The utility of real-time data analysis emerged as advantageous for infectious disease control amid the COVID-19 pandemic, aiding in limiting human contact, and facilitating trend analysis and demand forecasting. Despite the recognized benefits, concerns were raised regarding potential challenges associated with network and mobile data issues related to digitization. Recommendations included government investments in data-free applications, akin to banking apps, to address these challenges. Additionally, the strategic delegation of data capturing responsibilities to trained individuals, such as unemployed pharmacist assistants, was suggested to alleviate the workload on clinical personnel engaged in testing and reporting.

“Real-time data can be analysed remotely which is good for the control of infectious diseases like COVID-19 where human contact must be limited. It is also beneficial for establishing trends and forecasting future demand.”

“Digitization comes with network and mobile data issues therefore, the government must invest in the development of apps that do not need data to work, for example similar systems to bank apps.”

“The data capturing function should be allocated to data capturers in order to ease the burden on clinical personnel who have to do the testing and also report. These data capturers must be registered and trained to capture results on SVS. This is a good way to close the unemployment gap by roping in unemployed pharmacist assistants, people with inventory management skills.”

Advocacy for standardization was evident in proposals for the formulation and distribution of guidelines and manuals on inventory management by the National Department of Health.

“The National Department of health must create guidelines and manuals on inventory management and distribute them to all the relevant parties as a method to standardize data capturing across the healthcare facilities.”

Lastly, the emphasis on continuous monitoring and evaluation of the SVS by the National Department of Health was highlighted as vital for identifying areas of improvement and ensuring the optimal functionality and effectiveness of the system.

“To ensure that SVS is meeting its objectives it would be good for the National Department of Health to monitor and evaluate the system regularly to help in identifying areas for improvement and ensuring that the system is being used to its full capacity.

### 3.5 Visibility of information

This theme emerges prominently in participant comments, emphasizing the crucial role of collaborative information sharing in the context of pandemics like COVID-19. A participant highlighted the necessity for a unified response, emphasizing data, research findings, and implementable approaches exchanged among key stakeholders, including the National Department of Health, researchers, healthcare providers, and policymakers.

“Addressing pandemics such as COVID-19 requires a collaborative response and information sharing of data, research findings and approaches that can be implemented among the relevant stakeholders, for example National Department of Health, researchers, healthcare providers & policy-makers.”

Additionally, participants emphasized the significance of timely information dissemination by the National Department of Health across diverse platforms such as television and social media, utilizing multiple languages to enhance community comprehension. This proactive sharing of information was recognized as a potential deterrent against the propagation of misinformation within communities.

“Information needs to be disseminated timeously by the National Department of Health on various platforms, which includes our televisions and social media platforms. This information must in different languages for the communities to understand. If information is readily available, it will reduce the spread of fake news in our communities.”

Participants further noted the need for heightened visibility of COVID-19 information within healthcare facilities, expressing concern over the absence of pamphlets or posters, even during the pandemic.

“COVID-19 information must be visible. If you go into any health care facility now, there are no pamphlets or posters on COVID-19. Even during the pandemic, there were no posters available at our taxi ranks on where people can go and test.”

Recommendations were made to improve internal communication among stakeholders through regular meetings, ensuring comprehensive awareness and training on the significance of their contributions. These insights collectively underscore the pivotal role of information visibility in facilitating an effective pandemic response and fostering community engagement.

“Improve internal communication of stakeholders by meeting regularly where all stakeholders are kept well-versed and trained on the importance of their outputs.”

### 3.6 Procurement policies

In this theme, participants articulated the strategic imperative of establishing and standardizing procurement processes at a national level. Advocating for the leadership of the national government during pandemics, stakeholders emphasized the provision of standardized guidelines and policies for Supply Chain Management (SCM). Transparent communication, facilitated by the National Treasury through the dissemination of circulars and instruction notes detailing procurement processes to pertinent stakeholders, emerged as a crucial element. Participants expressed concerns about potential corruption and recommended the implementation of stringent procurement guidelines, favouring open tender systems over emergency procurement processes for enhanced accountability.

“When there is a pandemic, the national government has to lead in terms of building and guiding by providing standard guidelines and policies relating to SCM during a pandemic. The provinces can then build from what the national government have started.”

“The National Treasury should disseminate circulars and instruction notes to relevant stakeholders for transparency that outline the procurement processes that we need to follow and what we need to procure.”

“The National Treasury could have implemented strict procurement guidelines and processes to prevent corruption, such as the open tender system instead of using the emergency procurement system where there is no accountability.”

Additionally, participants stressed the importance of transparency in contract awards to qualifying and reputable suppliers, suggesting that such information should be accessible to the public. Proposals were put forth for the engagement of independent auditors to conduct regular audits on procurement processes within departments, with non-compliance carrying appropriate consequences. These insights collectively underscore the pivotal role of well-defined and transparent procurement policies in ensuring accountability, preventing corruption, and instilling public trust.

“Contracts must be awarded to qualifying and reputable suppliers and this information must be available to the public for transparency of the tender processes.”

“We can use independent auditors to conduct regular audits on the procurement processes in the department and those who do not comply must be taken to task.”

## 4 Discussion

This study, conducted through collaborative efforts with key stakeholders, identifies critical barriers in the supply chain of COVID-19 rapid tests and proposes priority areas for enhancing SCM of COVID-19 diagnostic services by co-creating a novel approach. The study emphasizes the significance of addressing barriers such as availability of testing kits, unknown demand, information on SCM during a pandemic, methods for controlling stock, and procurement in the context of the COVID-19 rapid test supply chain. Key stakeholders suggest a strategic focus on digitization, visibility of information, and effective procurement policies as integral components of this novel approach.

The literature corroborates the study’s findings on the crucial role of digitization in enhancing SCM processes. With technological advancements, there’s a notable shift from human-generated to machine-generated information (31). This entails a transition from traditional paper-based systems to digital supply chains, where sophisticated technologies such as RFID, Big Data, cloud computing, IoT, UAVs, and AI are assuming greater responsibility for data creation and management (32, 33). This transition offers substantial benefits, including enhanced efficiency and transparency (34). These emerging technologies enable organizations to develop self-optimizing and interconnected supply chain systems (35).

In this evolved landscape, the entire supply chain becomes integrated and intelligent, extending beyond the connection of patients, suppliers, and IT systems (36). It encompasses the inclusion of parts, products, and smart objects capable of real-time supply chain monitoring (37, 38). This is a supply chain system where every component, from raw materials to finished products, can communicate seamlessly. This level of connectivity and intelligence empowers organizations to forecast demand, optimize inventory levels, and swiftly adapt to market changes (39, 40). Ultimately, it fosters a more agile, efficient, and transparent supply chain ecosystem.

In these modern setups, sophisticated software harnesses data analytics and modeling to manage supply chains effectively (41). Instead of relying on manual processes and paper records, these systems leverage technology to track inventory, predict demand, and optimize logistics in real-time (42). This transformation equips organizations to make rapid decisions, respond promptly to changes, and enhance efficiency across the entire supply chain.

However, this study reveals that digitization at national, provincial, and local government levels is still in its early stages. The South African National Department of Health (NDoH) is pioneering a web-based application, the Stock Visibility System (SVS), at the PHC level, replacing paper-based tools. The SVS captures real-time data, acting as an early warning system and alerting stakeholders to PHC clinics with low stock levels, preventing potential supply interruptions (34, 43). Despite its effectiveness, the SVS does not yet include COVID-19-related commodities, hindering digital inventory management for these crucial supplies. Challenges identified, such as increased workload for staff and insufficient training, underscore the complexities of implementing digital solutions (40).

In addition to optimizing the SVS, effective procurement policies are essential for a robust pandemic response. These policies play a critical role in ensuring the availability and equitable distribution of essential medical supplies, equipment, and services to address health and economic consequences (44, 45). The standardization of SCM processes has been shown to provide consistency and uniformity in the SCM processes, leading to increased transparency, accountability, and quality of products and services (46–48). It also reduces costs and minimizes waste, while improving communication and coordination between different actors in the supply chain (49, 50).

Collaboration across different sectors, including government, healthcare systems, scientific communities, businesses, and civil society, emerges as a crucial factor in the context of the ongoing COVID-19 pandemic. The visibility of information about COVID-19 becomes paramount in promoting public health, preventing virus spread, and countering misinformation. Accessible and accurate information empowers individuals and communities to understand risks, take necessary precautions, and make informed decisions (51). It is also critical in combating misinformation and rumours about the virus, which can create panic and confusion among people. Misinformation can cause people to ignore public health guidelines, leading to an increase in infections and fatalities (52–55). Therefore, the visibility of information from trusted sources is crucial in providing accurate information and preventing the spread of misinformation (56). This collaborative effort is aligned with Sustainable Development Goal 3, emphasizing the need to strengthen health systems and improve access to health services during the pandemic.

### 4.1 Strengths and limitations of study

This study, notable for being the first to collaboratively co-create a novel approach for improving SCM of COVID-19 rapid tests in resource-limited settings, employs the NGT. The NGT, an innovative research method, ensures methodological rigor and facilitates collaboration with a diverse range of key stakeholders. Utilizing a virtual platform proves advantageous, allowing the inclusion of stakeholders from different sub-districts while minimizing travel disruptions. However, challenges related to network issues during virtual interactions due to load shedding were noted. The richness of ideas generated by the NGT, reflecting the multifaceted nature of SCM, posed a challenge in covering all identified barriers and strategies comprehensively.

### 4.2 Recommendations

Based on the study findings, several priority areas are recommended for the novel approach to improving SCM for SARS-CoV-2 diagnostic services:

- Enhancing the collaboration among key stakeholders; the government, non-governmental organizations, private sector suppliers, and research entities. Such collaborations will allow sharing of information and resources, research findings, best practices and improve distribution networks.
- Strengthening transparency and accountability across all levels of the supply chain through publicizing all procurement contracts and making them easily accessible to the public, ensuring that pricing is fair and transparent, and that suppliers are held accountable for meeting their commitments.
- Standardization of data capturing systems by South African NDoH across all healthcare facilities to ensure consistent and accurate data entry. This will help to ensure that the data collected is reliable and can be used for informed decision-making.
- Integrating the stock visibility system with other systems such as electronic health records, to enable a more comprehensive view of inventory levels and patient needs. This will allow healthcare providers to make more informed decisions about inventory management and patient care.
- Improve data analytics by adopting advanced data analytics tools such as machine learning and artificial intelligence. This will help to identify patterns and trends in inventory levels and demand, which can be used to optimize inventory management and improve supply chain efficiency.
- Continuous training of staff on how to use the stock visibility system effectively. This will help to ensure that the data collected is accurate and up-to-date, and that staff can use the system to make informed decisions about inventory management.
- Monitor and evaluate the SVS regularly to ensure that it is meeting its objectives. This will help to identify areas for improvement and ensure that the system is being used effectively.

## 5 Conclusion

In conclusion, this study sheds light on significant barriers in the supply chain of COVID-19 rapid tests and proposes a novel approach to improving SCM of COVID-19 diagnostic services. The study underscores the critical role of digitization, visibility of information, and effective procurement policies in enhancing SCM. While the South African National Department of Health is implementing the stock visibility system, the study advocates for the inclusion of COVID-19-related commodities in the system. Recommendations focus on collaboration, transparency, standardization, integration, data analytics, staff training, and ongoing monitoring and evaluation. Successful implementation of this novel approach necessitates collaboration across various sectors, including government, healthcare systems, scientific communities, non-governmental organizations, and civil society. A follow-up study is recommended to assess the feasibility of implementing these recommendations.

## Data availability statement

The original contributions presented in the study are included in the article/supplementary material, further inquiries can be directed to the corresponding author.

## Ethics statement

The studies involving humans were approved by University of Pretoria Faculty of Health Research Ethics Committee (Reference No: 655/2021, Dated: 24 November 2021) Limpopo Department of Health ethics committee (Reference No: LP_2021-12-007, Dated: 27 February 2022). The studies were conducted in accordance with the local legislation and institutional requirements. The participants provided their written informed consent to participate in this study.

## Author contributions

KM: Writing – original draft, Writing – review & editing, Conceptualization, Data curation, Formal analysis, Funding acquisition, Investigation, Methodology, Project administration, Resources, Software, Supervision, Validation, Visualization. AM: Supervision, Writing – review & editing. SN: Methodology, Writing – review & editing. BM: Methodology, Writing – review & editing. LM: Data curation, Writing – review & editing. NN: Data curation, Writing – review & editing. AL: Data curation, Writing – review & editing. NM: Data curation, Writing – review & editing. TM: Data curation, Writing – review & editing. TD: Data curation, Writing – review & editing. TM-T: Conceptualization, Methodology, Supervision, Writing – review & editing.
